# The Nonglycemic Actions of Dipeptidyl Peptidase-4 Inhibitors

**DOI:** 10.1155/2014/368703

**Published:** 2014-07-21

**Authors:** Na-Hyung Kim, Taeyang Yu, Dae Ho Lee

**Affiliations:** ^1^Hanbang Body-Fluid Research Center and College of Oriental Medicine, Wonkwang University, 460 Iksandae-ro, Iksan 570-749, Republic of Korea; ^2^Department of Internal Medicine, Wonkwang University School of Medicine & Hospital, 895 Muwang-ro, Iksan 570-711, Republic of Korea

## Abstract

A cell surface serine protease, dipeptidyl peptidase 4 (DPP-4), cleaves dipeptide from peptides containing proline or alanine in the N-terminal penultimate position. Two important incretin hormones, glucagon-like peptide-1 (GLP-1) and glucose-dependent insulinotropic peptide (GIP), enhance meal-stimulated insulin secretion from pancreatic *β*-cells, but are inactivated by DPP-4. Diabetes and hyperglycemia increase the DPP-4 protein level and enzymatic activity in blood and tissues. In addition, multiple other functions of DPP-4 suggest that DPP-4 inhibitor, a new class of antidiabetic agents, may have pleiotropic effects. Studies have shown that DPP-4 itself is involved in the inflammatory signaling pathway, the stimulation of vascular smooth cell proliferation, and the stimulation of oxidative stress in various cells. DPP-4 inhibitor ameliorates these pathophysiologic processes and has been shown to have cardiovascular protective effects in both *in vitro* and *in vivo* experiments. However, in recent randomized clinical trials, DPP-4 inhibitor therapy in high risk patients with type 2 diabetes did not show cardiovascular protective effects. Some concerns on the actions of DPP-4 inhibitor include sympathetic activation and neuropeptide Y-mediated vascular responses. Further studies are required to fully characterize the cardiovascular effects of DPP-4 inhibitor.

## 1. Introduction

In 2006, a new class of antidiabetic agents, dipeptidyl peptidase-4 (DPP-4) inhibitor, was approved for the treatment of type 2 diabetes mellitus [1, 2]. DPP-4 inhibitors (“gliptins” available as sitagliptin, saxagliptin, vildagliptin, linagliptin, and alogliptin) enhance meal-stimulated insulin secretion from pancreatic *β*-cells by sparing the hormone glucagon-like peptide-1 (GLP-1) and glucose-dependent insulinotropic peptide (GIP) from degradation by the enzyme DPP-4. Particularly, GLP-1 is a hormone produced by L-cells of the distal small intestine after ingestion of a meal [3]. In addition to the stimulation of insulin release, GLP-1 inhibits glucagon release, delays gastric emptying, and suppresses appetite [3]. GLP-1 and GIP are the so-called incretin hormones, which are involved in the higher insulin secretion induced by an oral glucose load compared to an equivalent intravenous glucose load. However, these two incretin hormones are degraded by DPP-4 within minutes after secretion [4]. Further detailed discussion about incretin hormones and incretin-based therapy involving GLP-1 is available from recent excellent reviews [3–7].

Besides GLP-1 and GIP there are more than 30 known peptide substrates for DPP-4 [8–10]. Therefore, DPP-4 inhibitor theoretically might increase the levels of an array of biologically active peptides* in vivo*. Before the use of DPP-4 inhibitors for the treatment of type 2 diabetes, DPP-4 had been intensively studied as an immune regulator because it acts as a T cell costimulator and a binding partner of adenosine deaminase (ADA) [9]. Furthermore, because of wide range of tissue distributions and other various functions of DPP-4, “gliptins” have been studied for their glycemic and nonglycemic actions. Under these backgrounds, this paper focuses on the nonglycemic actions of DPP-4 inhibitor. Many excellent reviews on the glycemic actions of DPP-4 inhibitors and incretins have been published recently and should be referred to for a more detailed review of these issues [6, 11].

## 2. DPP-4

DPP-4 (also known as CD26) was first described in 1966 by Hopsu-Havu and Glenner by its enzymatic activity in rat liver [12, 13]. This multifunctional type II transmembrane glycoprotein is a 110-kDa member of the prolyl oligopeptidase family, which functions as a cell surface serine protease, selectively cleaving dipeptides from peptides and proteins containing proline or alanine in the N-terminal penultimate (P_1_) position [9, 10]. This proteolysis by DPP-4-like activity can alter activities of target substrates, including the functional activation and inactivation of bioactive peptides or facilitated degradation of macromolecules by other peptidases [14].

A large cavity formed by the *α*/*β* hydrolase and the eight-bladed *β*-propeller domain acts as the substrate binding site [9, 10]. A catalytic triad of serine, aspartic acid, and histidine is found at the extracellular, C-terminal part of the molecule (Ser^631^, Asp^709^, His^741^ in the mouse sequence) [8]. Although peptides with proline and alanine in the penultimate position are exclusively accepted, those with other residues in the penultimate position also can be cleaved at low rates. Peptides with proline are far better hydrolyzed than the corresponding alanine-containing peptides. Optimal cleavage rates are generally observed at pH values between 7.5 and 8.5 [8]. A missense mutation of the DPP-4 gene in the catalytic site, namely, the substitution of glycine (633) to arginine, can cause the loss of activity. Due to this defect for active DPP-4, the DPP-4-deficient Fisher-344 rat strain has become available [47].

DPP-4 is widely expressed on T cells, B cells, natural killer cells, subsets of macrophages, hematopoietic stem cells, and hematopoietic progenitor cells, as well as on epithelial, endothelial, and acinar cells of a variety of tissues including, but not limited to, bone marrow, lung, spleen, pancreas, kidney, liver, and the intestines [8, 9].

Enzymatically active DPP-4 is a homodimer [10]. A soluble form of DPP-4 that lacks intracellular and transmembrane regions is present in body fluids such as serum/plasma, cerebrospinal fluid, synovial fluid, bile, and semen, presumably as a result of its release from many cell types, including lymphocytes, hepatocytes, and adipocytes [48, 49].

In addition, DPP-4 can heterodimerize with fibroblast activation protein *α* (FAP*α*) and associate with ADA, fibronectin, collagen, mannose 6-phosphate/insulin-like growth factor II receptor (M6P/IGFIIR), CD45, CXCR4, and plasminogen 2 [15].

## 3. DPP-4 Activity and/or Structure Homologues (DASH) 

In addition to DPP-4, there are several molecules with and without the same dipeptidyl peptidase activity which are structurally related to DPP-4 [48]. This family of proteins is known as the “DPP-4 activity and/or structure homologues” (DASH) [10, 14]. With enzymatic activity, DPP-4 and FAP*α* are located on plasma membrane, while DPP-8 and DPP-9 are located in cytoplasm. DPP-6 and DPP-10 are plasma membrane proteins homologous to DPP-4 without peptidase activity. In addition, DPP-7 is another homologous protein in intracellular vesicles, known as quiescent cell proline dipeptidase (QPP) or DPP-II, with DPP-4 activity. There is substantial overlap of substrate specificity and catalytic properties, which indicates the importance of this enzymatic activity, as well as the critical regulation of DASH expression and tissue specificity [12]. The important functions of each molecule in the DASH family are summarized in Table 1. Detailed description of each DASH molecule is beyond the scope of this review.

## 4. The Regulation of DPP-4

DPP-4 expression is influenced by hypoxia, and hypoxia-inducible factor-1*α* (HIF-1*α*) is a strong inducer of DPP-4 gene and protein [50]. Besides HIF-1*α*, hepatocyte nuclear factor-1*α* (HNF-1*α*), interferons, retinoic acid, and various cytokines have been shown to activate DPP-4 [51, 52]. The promoter of human DPP-4 gene contains putative binding sites for Sp1, AP-1/2, epidermal growth factor receptor-transcription factor site, HNF-1, signal transducer and activator of transcription 1*α*, and nuclear factor-*κ*B (NF*κ*B) [52].

In HepG 2 cells, DPP-4 activity was shown to be markedly increased by treatment with sodium butyrate, a histone deacetylase inhibitor [53]. In the rat intestine, DPP-4 can be induced by feeding a high-proline-containing gelatin diet [54]. However, DPP-4 can be regulated in a cell- or tissue-specific manner. In Caco2 cells, which belong to an epithelial intestinal cell line, high glucose concentrations suppress DPP-4 gene expression, resulting in decreased DPP-4 enzymatic activity; the glucose regulation of DPP-4 gene expression in Caco-2 cells is mediated by HNF-1*α* [55].

Interestingly, serum DPP-4 concentrations were significantly higher in apoE-deficient mice than C57BL/6 mice, and this difference increased with age [27]. Some studies suggested that glucose level affects DPP-4 activity and expression per se [51, 56, 57]. T cell DPP-4 expression, serum soluble DPP-4, and DPP-4 activities were shown to be increased in patients with type 2 diabetes [56]. Lower DPP-4 levels following exercise training plus weight loss were shown to be related to increased insulin sensitivity in adults with metabolic syndrome [58]. And, metformin, an antidiabetic agent, was demonstrated as a previously unrecognized DPP-4 inhibitor [59], although the mechanisms of its action are not entirely understood. Glypican-3, one of the six mammalian glypicans (heparin sulphate proteoglycans attached to the plasma membrane via a glycosyl phosphatidyl-inositol linkage), has been shown to inhibit DPP-4 activity in hepatocarcinoma cells and hematopoietic stem/progenitor cells [60–62]. Tissue factor pathway inhibitor (TFPI) binds to glypican-3 [61] and enhances glypican-3-mediated inhibition of DPP-4 [62].

DPP-4 release from various cells or tissues can be regulated by multiple factors: T cell by activation; differentiated adipocyte by tumor necrosis factor *α* (TNF*α*) or insulin; and endothelial cells by oxidative stress (H_2_O_2_) [63–65]. DPP-4 release into circulation seems to be decreased in some rheumatologic diseases, including rheumatoid arthritis (RA). In patients with RA, serum DPP-4 level and activity were decreased, while synovial fluid soluble DPP-4 level was similar to that of the controls [66]. Furthermore, in inflammatory bowel disease, serum DPP-4 activity showed an inverse correlation with known disease activity scores as well as with the concentrations of C-reactive protein and orosomucoid in serum [67]. These findings suggest that circulating DPP-4 may originate from various cells and tissues depending on disease state. Interestingly, plasma concentrations of DPP-4 protein increased after a single dose and after 12 weeks of treatment with the DPP-4 inhibitor, sitagliptin, in patients with type 2 diabetes, while plasma DPP-4 enzymatic activity decreased by more than 80% for the entire duration of DPP-4 inhibitor therapy [42]. The DPP-4 mRNA expression in peripheral blood mononuclear cells is suppressed by DPP-4 inhibitor [42]. The reason for the discrepancy between plasma protein level and enzymatic activity of DPP-4 still remains to be elucidated; the tissue origin of the high circulating DPP-4 after DPP-4 inhibitor therapy is not known. Further studies are warranted to determine the pathophysiologic relevance of circulating DPP-4 and its activity.

## 5. Enzymatic Substrates of DPP-4

Although the chain length of the peptides cleaved has not been systematically investigated, many peptides with N-terminal penultimate proline or alanine and up to 80 residues have been listed as substrates for DPP-4 (Table 2) [12, 48]. The *K*_*m*_ values of purified human DPP-4 for natural substrate peptides are in the micromolar range, while* in vivo* DPP-4 substrates act in the pico- or nanomolar range. Thus, the rate (specificity) constant *k*_cat_/*K*_*m*_ has been commonly used for comparing the potency of DPP-4 towards substrate peptides at physiological concentrations. Higher rate constants, corresponding to high cleavage rates at low and physiologic concentrations, are reported for neuropeptide-Y (NPY), peptide YY (PYY), and growth hormone-releasing hormone (GRH), compared to the value for GLP-1 or GIP [8]. However, studies about DPP-4 substrates other than GIP and GLP-1 are limited. Further studies will be required to elucidate the effect of DPP-4 inhibitor therapy on various substrates other than the well-known incretin hormones.

In the long list of DPP-4 substrates, NPY(1–36) and PYY(1–36) are of particular interest, because the *k*_cat_/*K*_*m*_ constants of DPP-4 for these neuropeptides are much higher compared with those for GLP-1 and GIP, respectively [8, 36]. NPY is an abundant neuropeptide in the central and peripheral nervous system; it is involved in the control of feeding, energy homeostasis, and blood pressure [68]. PYY(1–36) is released in proportion to nutrient intake along the gut and cleaved to PYY(3−36) by DPP-4. The ligand PYY(3–36) is selective for Y_2_ and has an anorexigenic effect [69]. Both NPY(1–36) and PYY(1–36) are potent endogenous agonists of the Y_1_ receptor, whereas enzymatically cleaved PYY(3–36) and NPY(3–36) are inactive at Y_1_ receptor but active at the Y_2_ and Y_5_ receptors. The Y_1_ receptor stimulates food intake, promotes vasoconstriction and cell proliferation, and is also involved in the regulation of heart rate, anxiety, and bone homeostasis [69]. The Y_2_ receptor is often found presynaptically, inhibiting the release of NPY and noradrenaline, and the Y_5_ receptor is abundant in the hypothalamus and involved in feeding behavior [8, 36]. Therefore, DPP-4 may divert the actions of these two neuropeptides from Y_1_ receptor-mediated actions to other Y receptor actions. Although* in vitro* cell culture experiments showed that DPP-4 inhibition enhanced Y_1_ receptor-mediated proliferation of preglomerular vascular smooth muscle cell and glomerular mesangial cells from spontaneously hypertensive rats,* in vivo* integrated responses to these peptides after chronic DPP-4 inhibitor therapy are not fully characterized yet [36]. The DPP-4-NPY(3–36)-Y_2_ receptor system was shown to have an important role in adipogenesis and angiogenesis in white adipose tissue [70].

Several members of CXC and CC chemokine subfamilies share a conserved Xaa-Pro or Xaa-Ala sequence at their N-termini which conforms to the substrate specificity of DPP-4 [8]. Integrated* in vivo* experiments that evaluate the relevance for DPP-4 as an important regulator of chemotactic responses and inflammation should be undertaken. Among the DPP-4 substrate chemokines, stromal cell-derived factor-1 (SDF-1) has been intensively studied. SDF-1, expressed as two different splice variants, SDF-1*α*(1–68) and SDF-1*β*(1–72), is a homing molecule for hematopoietic stem cells (HSCs), hematopoietic progenitor cells (HPCs), and endothelial progenitor cells (EPCs). SDF-1 is constitutively expressed by stromal cells in the bone marrow (BM) and in most organs, although an upregulation of its expression takes place after injury [18]. BM-derived EPCs can be mobilized into the blood stream in response to SDF1, which is released from damaged or ischemic vasculature. These EPCs are able to form a patch at sites of endothelial denudation and reconstitute the anatomical integrity of the intimal layer [51]. Thus, the interaction between SDF-1 from hypoxic tissues and its receptor CXCR4 on EPCs seems to promote vascular repair and neoangiogenesis. Interestingly, in addition to SDF-1*α*, CXCR4 is also upregulated by hypoxia-induced HIF-1 activation [18]. Some preclinical studies have shown that DPP-4 inhibition after acute myocardial infarction improves cardiac homing of stem cells and enhances heart function [71]. DPP-4 inhibitor therapy in patients with type 2 diabetes was shown to increase circulating SDF-1*α* and EPC levels [28]. Therefore, SDF-1*α* may contribute to one of pleiotropic effects of DPP-4 inhibitor with important implications for cardiovascular (CV) protection.

## 6. Immunologic and Inflammatory Actions of DPP-4 Inhibitor

DPP-4 is highly expressed in the membrane of a variety of cells including T cells, monocytes, and endothelial cells (9). For the stimulation of T cells through the CD3/T cell receptor complex, a costimulatory signal is required [72, 73]. Although its expression level is low in resting human lymphocytes, the expression is upregulated by stimulation [65]. DPP-4 triggering in T cells results in a series of events, such as phosphorylation of different proteins including the TCR/CD3zeta chain, IL-2 production, and T-cell proliferation [74]. Although the exact role of DPP-4 in this costimulatory pathway is not yet fully elucidated, it was reported that the interaction of dimeric DPP-4s cytoplasmic tail with CARMA1 [caspase-recruitment domain (CARD) membrane-associated guanylate kinase (MAGUK) protein 1] leads to NF*κ*B activation in T cells [75]. DPP-4 is also involved in the interaction between antigen-presenting cells (APCs) and T cells. The interaction of DPP-4 on T cells with caveolin-1 on APCs results in CD86 upregulation, therefore enhancing the subsequent interaction of CD86 and CD28 on T-cells to induce antigen-specific T-cell proliferation and activation [75]. DPP-4 enzyme activity seems to be required for the enhancement of T cell responses to various stimuli [73, 76, 77]. However, further studies will be required to establish the exact role of DPP-4 in immune processes, because many of previous studies were performed by using nonspecific DPP-4 inhibitors or cross-linking CD26 antibodies [78]. DPP-4 binds ADA. ADA is an enzyme responsible for the degradation of adenosine and deoxyinosine—molecules that inhibit the functions of lymphocytes. ADA binding is unique to DPP-4 among DASH family and DPP-4 inhibitor was reported not to affect this binding [14]. A previous study showed that a DPP-4 inhibitor inhibits ADA activity, leading to the suppression of monocyte migration [79].

Research interests regarding DPP-4 have been recently extended into monocytes, macrophages and dendritic cells [80]. Similarly, recent studies have demonstrated the effect of DPP4 inhibitors on the reduction of proinflammatory cytokines in macrophages, visceral adipose tissue, and atherosclerotic plaques [80, 81].

Some studies showed anti-inflammatory effects of selective DPP-4 inhibitors in patients with type 2 diabetes [42, 82], while others failed to prove the effects [83]. And, it has yet to be resolved whether the various anti-inflammatory properties of DPP-4 inhibitor will translate into improved clinical outcomes in diabetic patients.

## 7. Other Actions of DPP-4 Inhibitor

Sitagliptin was shown to inhibit platelet aggregation via its inhibitory effects on thrombin-induced rise in concentration of intracellular free calcium and on thrombin-induced tyrosine phosphorylation of multiple proteins in human platelets [44]. However, Krijnen et al. [32] observed a marked decrease of microvascular endothelial DPP-4 expression in recently infarcted human hearts. This finding suggests that DPP-4 has an antithrombotic effect. And, via cleaving N-terminal Gly-Pro from the fibrin *α*-chain, DPP-4 can inhibit fibrin polymerization and clot formation [84]. Moreover, treatment of human umbilical cord vein endothelial cells (HUVECs) with diprotin A, a DPP-4 inhibitor, increased the expression of endothelial tissue factor and consequently induced adherence of platelets to the ECs, although platelet aggregation was not increased [32].

An elegant study using labeled cholesterol in a mouse model showed that sitagliptin promotes reverse cholesterol transport through reduced intestinal cholesterol absorption [85]. And, vildagliptin was shown to decrease the level of hepatic mRNA transcript for farnesyl di-phosphate transferase in dual incretin receptor knockout mice [86]. Farnesyl di-phosphate is a substrate for the synthesis of dolichol, coenzyme Q10, and cholesterol. Although previous studies suggested that the postprandial lipid lowering effect of DPP-4 inhibitor is dependent on GLP-1 [87], DPP-4 inhibition may have a direct effect on cholesterol metabolism by affecting the isoprenoid pathway, especially in cases of vildagliptin and alogliptin [88, 89].

GLP-1 decreases Na^+^/H^+^ exchanger (NHE3)-mediated sodium reabsorption in rodent experiments [90]. The DPP-4 inhibitor alogliptin administered in metabolic cage studies increased urinary excretion of sodium in both wild type and GLP-1 receptor-deficient mice, indicating that the DPP-4 inhibitor has GLP-1-dependent and -independent natriuretic effects [90].

## 8. Cardiovascular Effects of DPP-4 Inhibitor

The risk of CV disease is two to four times as high in subjects with diabetes as in subjects without diabetes. DPP-4 inhibition has a small but significant blood pressure- (BP-) lowering effect, although this effect may be dependent on the model of hypertension [30, 91]. Many studies have shown that treatment with DPP-4 inhibitor improves endothelial function in patients with type 2 diabetes [92], in both GLP-1-dependent and -independent manners [7]. In preconstricted aortic segments from C57BL/6 mice, alogliptin treatment resulted in dose-dependent vasorelaxation through a GLP-1 receptor-independent, Src-Akt-endothelial nitric oxide synthase pathway [79]. In a recent study, it was reported that DPP-4 inhibitor treatment led to a reduction of lipid and protein oxidation in a rat model of renovascular hypertension [30]. DPP-4 can bind M6P/IGF-IIR which functions in two distinct biological processes; protein trafficking and transmembrane signal transduction [93]. It was already reported that the DPP-4 and M6P/IGF-IIR interaction contributes to T cell activation [14]. DPP-4 can directly act on HUVECs to stimulate reactive oxygen species (ROS) generation and RAGE [receptor for advanced glycation end products (AGE)] gene induction via the interaction with M6P/IGF-IIR [64]. Furthermore, linagliptin inhibited the AGE-induced soluble DPP-4 production, ROS generation, and gene expression levels of RAGE, intercellular adhesion molecule-1, and plasminogen activator inhibitor-1 in HUVECs [64]. Additionally, soluble DPP-4 stimulates the proliferation of cultured vascular smooth muscle cells (VSMCs) while DPP-4 inhibitor suppresses the proliferation by inhibiting ERK phosphorylation [27]. It was reported that treatment with des-fluoro-sitagliptin, a DPP-IV inhibitor, reduced restenosis in obese type 2 diabetic rats following balloon injury to the carotid artery [94]. The study also revealed that des-fluoro-sitagliptin treatment suppressed proliferation of VSMCs, promoted apoptosis of VSMCs and reduced inflammatory process and MMP-2 and MMP-9 expressions in the injured artery [94].

Short-term treatment with a DPP4 inhibitor, vildagliptin, was shown to prevent left ventricular hypertrophy caused by continuous infusion of isoproterenol. These effects were accompanied by the amelioration of perivascular fibrosis and expression of genes associated with glucose uptake (GLUT4) and inflammation (TNF*α* and IL-6) [23].

In the kidneys of diabetic mice, the DPP-4 protein levels were upregulated as compared with control kidneys. Both glomerulosclerosis and tubulointerstitial fibroses occurring in the diabetic kidney are associated with increased DPP-4 protein and activity and increased transforming growth factor-*β*2 signaling [95]. Linagliptin was shown to ameliorate all of above-mentioned pathophysiologic changes in the diabetic kidney [95]. Many studies strongly support the antiatherosclerotic and CV-renal protective effects of DPP-4 inhibitor [79]. Recent meta-analysis of clinical trial data have shown more favorable CV outcomes with DPP-4 inhibitors than with other classes of antidiabetic agents [91, 96, 97].

However, in two recent randomized controlled studies [the Examination of Cardiovascular Outcomes with Alogliptin versus Standard of Care (EXAMINE) trial and The Saxagliptin Assessment of Vascular Outcomes Recorded in Patients with Diabetes Mellitus (SAVOR) Thrombolysis in Myocardial Infarction (TIMI) 53 trials], DPP-4 inhibitor therapy in high risk patients with type 2 diabetes did not show a CV protective effect [98, 99]. No obvious explanation is available currently to explain the neutral, rather than protective, effects on CV outcomes in these clinical trials. More information is needed on the various effects of DPP-4 inhibitors. Various nonglycemic actions of DPP-4 inhibitors are summarized in Table 3.

**Figure 1:**
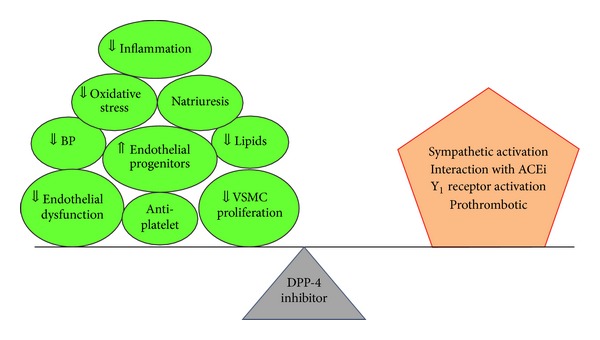
Nonglycemic actions of DPP-4 inhibitor in relation with pathophysiology of CV disease. Despite its many salutary effects on the CV system, DPP-4 inhibitor therapy in patients with type 2 diabetes and CV disease did not show a secondary prevention effect. Some unfavorable actions of DPP-4 inhibitor need to be further characterized to improve DPP-4 inhibitor therapy in patients with type 2 diabetes. ACEi, ACE inhibitor.

Finally, some of unfavorable actions of DPP-4 inhibitors need to be addressed in this paper (as described in italics in Table 3). In an interesting study, DPP-4 inhibition lowered BP during acute administration of the low dose angiotensin-converting enzyme (ACE) inhibitor enalapril, but abolished the acute antihypertensive effects of high dose enalapril in patients with metabolic syndrome [31]. In a recent study, when vildagliptin was administered to cynomolgus monkeys at high dose, skin lesions on the distal extremities (hands, feet, ears, and tail) appeared after three weeks of treatment and consisted of blister formation, peeling and flaking skin, erosions, ulcerations, scabs, and tail sores. These lesions were mediated by endothelial and medial hypertrophy/hyperplasia of arterioles at various levels of the dermis. These pathologic changes were related to increased NPY-Y_1_ receptor signaling [33]. In humans, Boschmann et al. showed that vildagliptin administration increased plasma norepinephrine (NE) concentrations in response to meals without causing a change in epinephrine levels [38]. Use of DPP-4 inhibitors may cause a small increase in resting heart rate as well as plasma NE when used in conjunction with a high-dose of the ACE inhibitor enalapril [31]. DPP-4 may have more roles in the inactivation of substance P when ACE is inhibited. Substance P acts as a vasodilator but also increases sympathetic outflow [100]. In a recent human study, substance P-stimulated heart rate and sympathetic activity (as assessed by venous plasma NE) was significantly higher during combined ACE and DPP-4 inhibition than during DPP-4 inhibition alone [100]. In addition, DPP-4 inhibition diminished substance P-induced tissue plasminogen activator release in women [100]. DPP-4 inhibition causes arterial PYY(1–36) and NPY(1–36) to enhance Angiotensin II- (Ang II-) induced renal vasoconstriction more effectively in genetically susceptible kidneys [39]. This finding strongly suggests that renovascular DPP-4 inactivates NPY(1–36) so that low concentrations cannot enhance the renovascular effects of Ang II. However, when DPP-4 is inhibited, this inactivation is impaired and even low concentrations of NPY(1–36) may potentiate renovascular responses to Ang II. Taken together, DPP-4 inhibition in certain conditions may cause sympathetic activation and selective enhancement of the NPY-Y_1_ receptor pathway, leading to vasoconstriction and BP elevation. Further studies are required to determine whether some of these unfavorable effects translate into negative CV outcomes (Figure 1).

## 9. Conclusions

DPP-4 inhibitor is a new class of oral antidiabetic drugs which, by inhibiting the degradation of GLP-1 and GIP, improves fasting and postprandial hyperglycemia. However, its target, DPP-4, has a wide range of biologic functions, in addition to its action on the incretin hormones. DPP-4 inhibitor has been shown to have pleiotropic effects and many studies have revealed its salutary CV actions. However, randomized clinical trials failed to prove potential CV protective actions of DPP-4 inhibitor in patients with type 2 diabetes. Although DPP-4 inhibitor has been shown to have anti-inflammatory and antiatherosclerotic effects, the drugs also seem to activate the sympathetic nervous system and cause selective enhancement of the NPY-Y_1_ receptor pathway. And, there is a plethora of DPP-4 substrates and DPP-4-interacting molecules. Further studies are required to fully characterize the nonglycemic effects of DPP-4 inhibitor, as knowledge on DPP-4 and its homologues expands.

## Figures and Tables

**Table 1:** The important functions of DASH molecules.

Molecules	Functions	Soluble form in the blood
DPP-4	Type II membrane glycoprotein with dipeptidyl peptidase activity	Yes

FAP (Seprase)	(i) Type II membrane glycoprotein with dipeptidyl peptidase, collagenase, and gelatinase activity (ii) Involved in extracellular matrix degradation, tissue remodeling, and fibrosis [15] (iii) Can heterodimerize with DPP-4 for efficient tissue remodeling [16]	Yes

DPP-7 (QPP, DPP-II)	(i) Intracellular location with dipeptidyl peptidase activity (ii) Most of its physiological substrate is unknown (iii) Secreted in an active form in response to calcium [17] (iv) Regulation of apoptotic pathway in quiescent lymphocytes [17]	Unknown

DPP-8/DPP-9	(i) Intracellular localization with dipeptidyl peptidase activity (ii) Seems to be involved in M1 macrophage activation [18] (iii) Up-regulation of DPP-9 during monocyte to macrophage differentiation [18] (iv) Can cleave releasable neuropeptide Y (NPY) in brain [19] (v) The regulation of cellular proliferation and apoptosis [19]	No

DPP-6 and DPP-10	(i) Transmembrane proteins with no peptidase activity (ii) Binds to specific voltage-gated K^+^ channels, altering their structures and biophysical properties [20]	No

**Table 2:** Possible enzymatic substrates of DPP-4.

Regulatory peptides	Brain natriuretic peptide, GIP, gastrin-releasing peptide (GRP), GLP1, GLP2, GRH, pituitary adenylate-cyclase-activating polypeptide (PACAP)-(1–38), vasoactive intestinal peptide (VIP)

Chemokines	Eotaxin (CCL11), IP10 (CXCL10), I-TAC (CXCL11), macrophage-derived chemokine (MDC, CCL22), monokine induced by gamma-interferon (CXCL9), RANTES (CCL5), stromal cell-derived factor-1 (CXCL12), monocyte chemotactic protein-2, granulocyte chemotactic protein-2

Neuropeptides	NPY(1–36), substance P, PYY(1–36), bradykinin, endomorphin-2

Others	Granulocyte macrophage-colony stimulating factor (GM-CSF) G-CSF, erythropoietin, Interleukin-3, fibroblast growth factor-2, thrombopoietin

IP, interferon-*γ*-inducible protein; I-TAC, Interferon-inducible T cell a chemoattractant; RANTES, regulated on activation normal T cell expressed and secreted.

**Table 3:** Various non-glycemic actions of DPP-4 inhibitors.

Tissues/systems	Effects of DPP-4 inhibitor
Heart	(i) Reduce infarct size after myocardial ischemia/reperfusion injury [21] (ii) Decrease cardiac fibrosis in uremic cardiomyopathy model [22] (iii) Prevent left ventricular hypertrophy caused by continuous infusion of isoproterenol [23]

Vascular systems	(i) Decrease RAGE expression [24] (ii) Vascular relaxation and increase nitric oxide release [25] (iii) Reduce atherosclerotic lesion [26] (iv) Attenuate soluble DPP-4-induced VSMC proliferation [27] (v) Increase the number of circulating EPCs and plasma level of SDF-1*α* in type 2 diabetic patients [28] (vi) Neuroprotective in ischemic stroke model [29] (vii) Reduce lipid and protein oxidation [30] (viii) *Abolish the BP-lowering effects of enalapril in patients with metabolic syndrome *[31] (ix) *Prothrombotic effect *[32] (x) *Y*_1_ * receptor-mediated endothelial and medial hypertrophy/hyperplasia of arterioles in a specific condition *[33]

Kidney	(i) Decrease NaHCO_3_ reabsorption in renal proximal tubule by inhibiting Na^+^/H^+^ exchanger type 3 activity [34] (ii) Inhibit glomerulosclerosis, fibrosis, and albuminuria [35] (iii) *Stimulate extracellular matrix production in mesangial cells mediated by Y*_1_ * receptor activation *[36]

Liver	Improve hepatic steatosis [37]

Neuro-endocrine systems	(i) *Increase plasma norepinephrine after meals *[38] (ii) *Enhance Y*_1_ * receptor-mediated renovascular responses to Angiotensin II in kidneys from genetically-susceptible kidneys *[39]

Immune systems	(i) Suppress MMP-1, proliferation and some cytokine production (IL-6, IL-1*β*) in histiocytic cell lines [40, 41] (ii) Anti-inflammatory actions [42] (iii) Attenuation of ischemia/reperfusion injury in lung transplants in association with increased levels of intrapulmonary VIP [43] (iv) May inhibit the cytopathic effects of HIV-1 [8]

Hematopoietic system	(i) Anti-platelet effect [44] (ii) Increased recovery of hematopoiesis after bone marrow suppression (iii) Expansion of HPCs [45, 46]

Note: presumed problematic actions that may cause adverse CV effects are *in italic*.
